# The Role of Surfactant in Electrocatalytic Carbon Dioxide Reduction in the Absence of Metal Cations

**DOI:** 10.1021/acselectrochem.4c00040

**Published:** 2024-10-03

**Authors:** Hansaem Jang, Adrian M. Gardner, Lucy J. Walters, Alex R. Neale, Laurence J. Hardwick, Alexander J. Cowan

**Affiliations:** † Stephenson Institute for Renewable Energy (SIRE) and the Department of Chemistry, University of Liverpool, Liverpool L69 7ZF, United Kingdom; ‡ Low Energy Ion Scattering Facility, George Holt Building, University of Liverpool, Brownlow Street, Liverpool L69 3GB, United Kingdom

**Keywords:** CO2RR, eCO2R, SEIRAS, SEIRA, CTAB

## Abstract

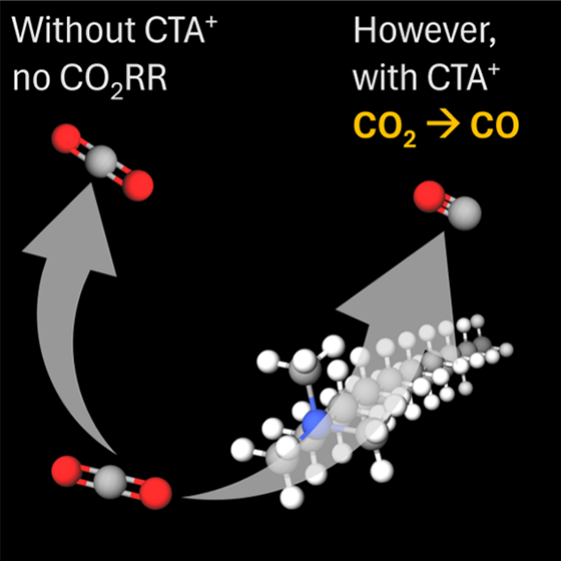

Carbon dioxide electroreduction does not occur on Au when metal cations are absent from the electrode surfaces. Here we show that the electroreduction can be enabled without metal cations, albeit with low efficiency, by the presence of cationic surfactants on Au. The findings demonstrate that in addition to possibly stabilizing CO_2_ reduction intermediates the presence of surfactants plays a role in suppressing the competing reactions. At potentials negative of a critical potential, a cationic surfactant adsorbs onto the electrode surface, displacing interfacial water molecules, hampering the access of proton donors to the electrode surface and inhibiting hydrogen evolution during electrolysis.

Carbon dioxide can be electrochemically reduced into CO on Au with a high Faradaic efficiency (FE) and the CO_2_ reduction reaction (CO_2_RR) occurs only when cations other than H^+^ are added to the electrolyte.^1−4^ The presence of alkali metal cations is proposed to be vital to coordinatively stabilize initially bound CO_2_, thus enabling CO_2_RR. However, the use of alkali metal cations in CO_2_ electrolyzers can cause “salting out” effects, where carbonate salts precipitate during electrolysis, blocking gas channels and impeding the long-term operation.^5,6^ Instead of metal cations, a small number of organic cations and cationic functionalities have been shown to enable the CO_2_RR at the electrode surface.^7−11^

Surface additives, such as the surfactant cetyltrimethylammonium bromide (CTAB), have been used to synthesize Au catalysts and to tune the activity of CO_2_RR in the presence of metal cations.^12−14^ CTAB has been shown to modify the product distributions of CO_2_RR and minimize the competitive hydrogen evolution reaction (HER).^15−19^ CTAB has been proposed to adsorb on the electrode upon cathodic polarization, displacing water from the electrode surface, stabilizing reduced carbon species, changing interfacial electric fields, and modifying the surface structure.^18−25^ However, the possibility of the cationic functionality of cetyltrimethylammonium (CTA^+^) enabling CO_2_RR in the absence of other (e.g. alkali metal) cations has not been explored despite a small number of emerging reports that organic cations may be able to take on this role.^7,8^

Here we perform electrolysis (Figure S1) in acidic environments (1 mM H_2_SO_4_) where cations other than H^+^ are absent in the electrolyte and explore if the addition of CTAB can enable CO_2_RR. We first attempt to validate the hypothesis electroanalytically, based on the methodology reported by Koper et al.,^4^ where CO produced by CO_2_RR is identified by the CO-stripping during a cyclic voltammetry (CV) experiment. In Figure 1, all panels have a reduction peak at ca. –0.3 V versus the reversible hydrogen electrode (RHE; hereinafter all potentials are referenced against RHE). Under CO_2_ when 1 mM K_2_SO_4_ is added to 1 mM H_2_SO_4_ electrolyte (Figure 1a, Red), an increase in current is seen between −0.65 and −0.8 V compared to experiments under Ar (Figure 1a, Black), assigned to a combination of CO_2_RR and HER. A thorough discussion of the regions (R1–R4) of the voltammogram is given in the later mechanistic discussion.

**Figure 1:**
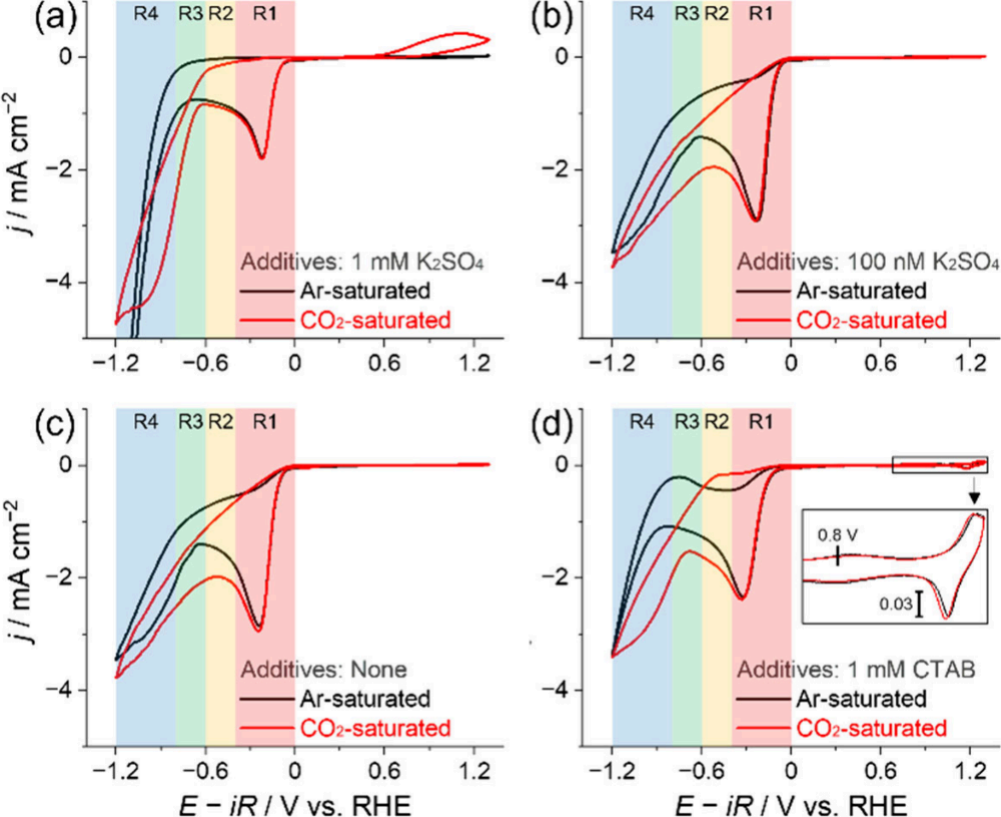
Cyclic voltammograms obtained on Au using 1 mM H_2_SO_4_ electrolyte with (a) 1 mM K_2_SO_4_, (b) 100 nM K_2_SO_4_, (c) no additives, and (d) 1 mM CTAB. The electrolyte was purged and saturated with (Black) Ar or (Red) CO_2_. The electrode potential was initially scanned from the OCP to −1.2 V (potentials negative of CO evolution), then to 1.3 V (potentials positive of CO oxidation or CO-stripping), and back to the OCP at 50 mV s^–1^.

Past studies have shown that alkali metal cation concentrations on a millimolar scale can enable the CO_2_RR.^4^ Using 1 mM K_2_SO_4_ electrolyte (Figure 1a), we confirm the CO_2_RR to produce CO through the presence of a notable CO-stripping peak at potentials positive of 0.7 V. When [K^+^] is on a nanomolar scale, a CO-stripping peak is unseen (Figure 1b). In the H_2_SO_4_ electrolyte devoid of any further cations (Figure 1c), the CV shows no sharp increase in current under CO_2_ at potentials negative of –0.65 V and only capacitive features at potentials positive of 0.7 V where CO-stripping would be expected to occur. It confirms that in the absence of K^+^, no CO_2_RR takes place, in agreement with the past reports.^4^

With CTAB present in the electrolyte (Figure 1d), we observe a sharp increase in current at potentials negative of –0.65 V under CO_2_, indicating possibly enabled CO_2_RR. Attempts to measure CO electroanalytically by CO-stripping when CTAB is present are complicated as anodic peaks arise at potentials near 0.8 and 1.25 V (Figure 1d inset, Figure S2). This potential window coincides with the characteristic signatures of CTA^+^,^26^ as well as CO-stripping. To decouple these two features, we conducted electrolysis in CTAB-containing electrolyte saturated with Ar, by which only the HER was allowed to proceed without the evolution of CO during the electrolysis. Under this condition, the CV shows anodic peaks again at the same potentials, i.e. 0.8 and 1.25 V, with the same intensity in current (Figure S2). Therefore, the observed anodic peaks should be assigned to CTA^+^ rather than to CO-stripping.

Figure 1d suggests that CO_2_RR may be occurring and that the presence of 1 mM CTAB may prevent CO-stripping on Au. Surface enhanced infrared absorption spectroscopy (SEIRAS) experiments in the presence of CTAB at a thin-film Au electrode have been carried out to further explore the system (Figures S3, S4). The positive peaks at ca. 2100 and 2160 cm^–1^ observed in the spectra in the presence of CTA^+^ (Figure 2) are assigned to the accumulation of CO_ad_ on the Au surface, and potentially solvated CO close to the electrode surface, based on the literature.^18,27^ The absence of those peaks in the absence of CTA^+^ (Figure S5) indicates that the CO_2_RR only occurs when CTAB is present. Importantly, CO_ad_ was observed with the greatest intensity between 0.25 and 0.65 V, with a negligible frequency shift (Note S1; Figure S6). Given that CO-stripping occurs at 0.7 V (Figure 1a), the dramatic decrease of CO_ad_ at potentials positive of 0.65 V can be attributed to the electrooxidative removal of CO from the surface. Past studies have shown that CTA^+^ reorientates at potentials negative of the potential at the point of zero charge (*E*_pzc_) and adsorbs onto the electrode with the ammonium head group facing the electrode surface.^18,28^ Our SEIRAS experiment shows that CO_ad_ appears at potentials positive of the *E*_pzc_ (measured at 0.2 V in Figure S7) and this correlates with a decrease in the intensity of the ν(CH) modes of CTA^+^ (Figure S8, Note S2). The SEIRAS studies show that CO accumulation on Au is prevented by adsorbed CTA^+^. However, when the electrode is held at potentials positive of *E*_pzc_, disruption of the CTA^+^ layer allows a concentration of CO to bind to the surface in a limited potential window. During the CV measurements (Figure 1), the potential is rapidly changed; the potential window where CO is able to access the surface is limited and this, coupled to the time scale of reorientation of CTA^+^ and CO diffusion to the surface, prevents the observation of the CO_ad_ stripping peak.

**Figure 2:**
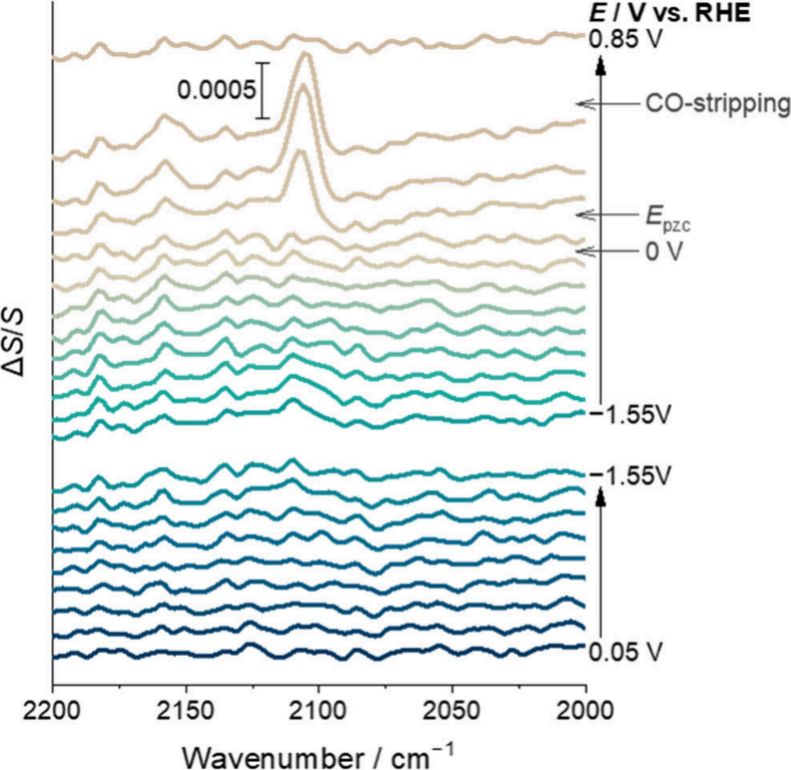
*Operando* SEIRAS spectra obtained in the 1 mM H_2_SO_4_ and 1 mM CTAB electrolyte saturated with CO_2_, as a function of varied applied potential in stepwise decrement or increment of 0.2 V. Baseline subtraction was applied and the reference spectra were taken at the open circuit; for details, see the Supporting information.

Bulk electrolysis in the presence of CTAB also confirms that CO_2_RR occurs when CTAB is present in the H_2_SO_4_ electrolyte with a FE_CO_ of 10.8 ± 3.4% (Figure S9). In contrast, in the absence of CTAB, a trace FE_CO_ of 0.38 ± 0.34% is determined. We note this is a relatively low FE for CO_2_RR in the CTAB experiment; but the aim here is to carry out an electrochemical study that demonstrates the role that CTA^+^ plays in CO_2_RR, which requires exclusion of other cations (except H^+^), and thus this mechanistic study necessitates operation in acidic solution, i.e., HER-dominating conditions. A possibility that CO evolved as a result of adventitious impurities was disproved by electroanalysis, gas analysis, and low-energy ion scattering spectra (Note S3; Figures S10–S15, Table S1). Another possibility that CO resulted from the electrolytic decomposition of CTA^+^ was also disproved (Note S4; Figure S16).

To understand the role of CTAB in the CO_2_RR, we now examine the behavior of competing reactions during electrolysis. For brevity, the potential windows of Figures 1 and 3 are divided into four regions: R1 (0 to −0.4 V), R2 (−0.4 to −0.6 V), R3 (−0.6 to −0.8 V), and R4 (−0.8 to −1.2 V). As described above under CO_2_ when either 1 mM of K_2_SO_4_ or CTAB is present (Figure 1a and 1d, Red), CO_2_RR takes place with the onset of –0.65 V within R3. CO_2_RR cannot occur under Ar but only the HER proceeds with two different proton donors: H^+^ and H_2_O. In the electrolytes used in Figure 1, the concentration of H^+^ (1.87 mM) is significantly lower than that of H_2_O (55 M). Therefore, the obtained limiting current peak within R1 (Figure 1a–1d, Black) is assignable to the electroreduction of H^+^, and the exponential increase in current within R4 is to the electroreduction of H_2_O, in-line with past studies.^1,29^

**Figure 3:**
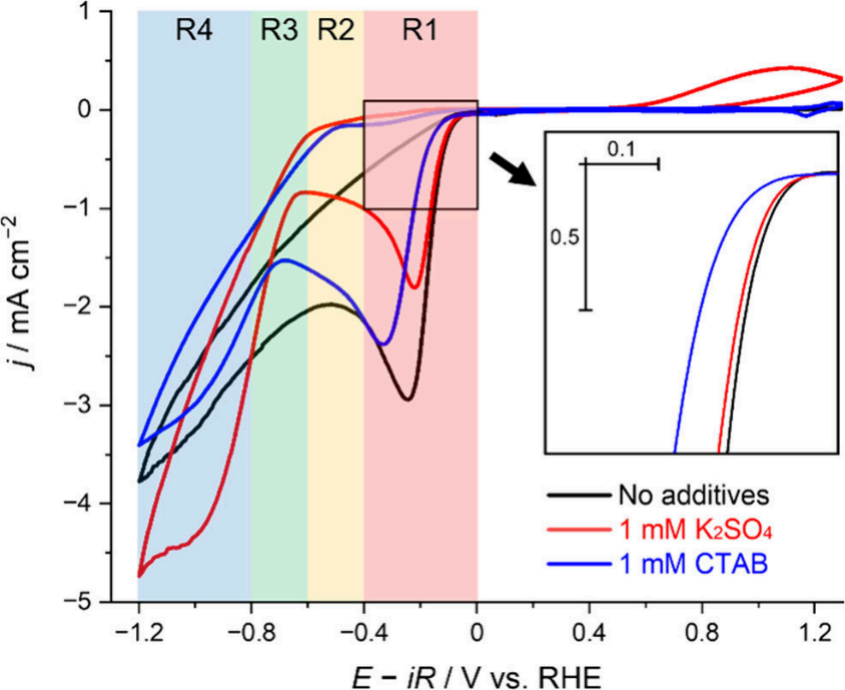
Cyclic voltammograms obtained on Au in CO_2_-saturated electrolytes using 1 mM H_2_SO_4_ electrolyte with additives, as detailed in the legend. Electrolytes: (Black) 1 mM H_2_SO_4_ alone without additives, (Red) 1 mM H_2_SO_4_ with 1 mM K_2_SO_4_, (Blue) 1 mM H_2_SO_4_ with 1 mM CTAB. The electrode potential was initially scanned from the OCP to −1.2 V (potentials negative of CO evolution), then to 1.3 V, and back to the OCP at 50 mV s^–1^.

CVs in 1 mM H_2_SO_4_ alone show a mismatch in the current between Ar and CO_2_ experiments throughout R2 (Figure 1c). HCO_3_^–^ has been proposed to serve as a proton donor at potentials negative of −0.43 V,^29^ and the increased current density under CO_2_ between −0.4 to −0.6 V (i.e. R2) is proposed to be due to the HER caused by HCO_3_^–^ (or HCO_3_^–^/H_2_). Essentially, the pH of 1 mM H_2_SO_4_ (i.e. 2.73) is too acidic to convert CO_2_ into HCO_3_^–^ via the carbonic acid system equilibrium at appreciable quantities in the bulk;^30^ however, the local pH at the electrode surface in R2 is expected to be higher than that in R1 as H^+^ are consumed during R1 by H^+^/H_2_. Therefore, we postulate that HCO_3_^–^ can exist in R2 and can work as a proton donor (Note S5; Figure S17). The contribution of HCO_3_^–^ to the HER appears to be lowered by high concentration metal cations (e.g., 2 mM [K^+^]), as the Ar-saturation CV overlaps the CO_2_-saturation CV in R2 (Figure 1a).

We speculated that the foregoing phenomenon is in part a consequence of the “shielding effect”. Metal cations at the electrode surface can shield the electric field, disrupting the diffusion of H^+^.^31^ When H^+^ transport decreases due to the shielding effect, H^+^ consumption within R1 will decrease, giving rise to a drop in H^+^/H_2_ current. As a result, in Figure 3, the limiting current in R1 drops from –2.85 mA cm^–2^ (for 1 mM H_2_SO_4_ alone) to –1.77 mA cm^–2^ (for 1 mM H_2_SO_4_ with 1 mM K_2_SO_4_). When the H^+^ consumption decreases, the local pH will increase less, converting a lower amount of CO_2_ into HCO_3_^–^. Consequently, when the electrode is under the shielding effect, the concentration of HCO_3_^–^ during R2 will decrease and the HCO_3_^–^ will contribute less to the HER, thereby decreasing the current (Note S6; Figure S18).

However, we have noted that a change in H^+^/H_2_ cannot be accounted for solely by the shielding effect (Note S7; Figure S19). We postulate that the suppressed H^+^/H_2_ activity in CTAB experiments also arises from the hydrophobic tail of CTA^+^ in addition to any shielding effect caused by its cationic functionality. The SEIRAS experiment shows that the interfacial water within the double layer is displaced by CTAB adsorption (Figure S8, Note S2), in-line with past studies.^18^ Once CTA^+^ is adsorbed and orientated at the interface, the arrival of H_3_O^+^ to the electrode surface through the electrolyte will be impeded by steric and hydrophobic effects. Figure 4 shows that 1 mM CTAB in solution under Ar shifted the H_2_O/H_2_ onset potential by–269 mV, when compared to the 1 mM H_2_SO_4_ electrolyte alone (Note S8; Figure S20). A shift of –131 mV is also measured in the 1 mM H_2_SO_4_ and 1 mM K_2_SO_4_ solution. A significantly larger increase in H_2_O/H_2_ overpotential when CTA^+^ is introduced indicates that the presence of CTA^+^ hampers the access of H_2_O to the electrode surface, more effectively than K^+^. Therefore, on the basis of both the electroanalytical and spectroscopic studies, it is rational to conclude that the hydrophobic effect plays a major role in disrupting H_2_O access to the electrode surface.

**Figure 4:**
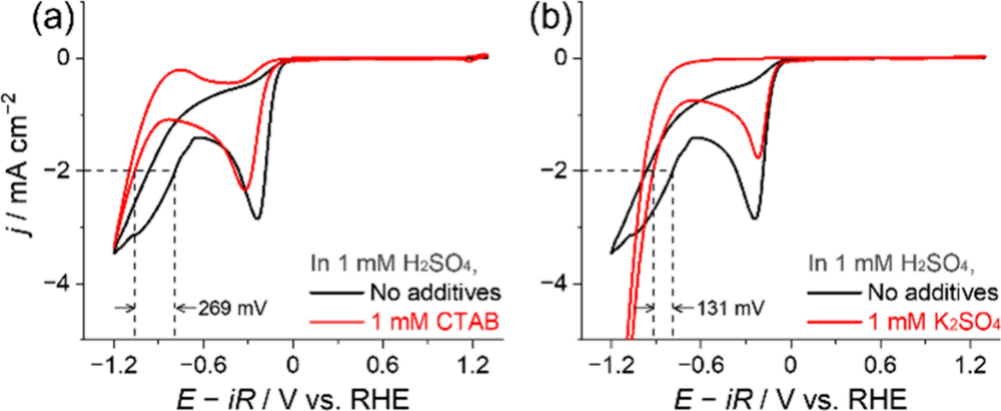
Cyclic voltammograms obtained on Au in Ar-saturated electrolytes, with or without (a) 1 mM CTAB or (b) 1 mM K_2_SO_4_ in the 1 mM H_2_SO_4_ solution. The electrode potential was initially scanned from the OCP to −1.2 V, then to 1.3 V, and back to the OCP at 50 mV s^–1^.

In summary, we have electroanalytically and spectroelectrochemically demonstrated the role of CTAB during electrolysis in the absence of metal cations. We showed that the presence of CTA^+^ inhibited the occurrence of competing reactions, including HER caused by H^+^ or H_2_O. These competing reactions were suppressed by a combination of the shielding effect and the hydrophobic effect with the latter being a dominant contribution. In addition, the CVs obtained under CO_2_-free conditions suggested that the local pH at the electrode surface could increase at potentials more negative than the H^+^/H_2_ onset potential, leading to a change in carbonic acid equilibria. We also note that the CO_2_RR enabled by surfactants is not unique to CTAB (Note S9), which suggests that the understanding of this study can facilitate and accelerate future developments.

## Supporting Information Available

Experimental details; cell configuration; comparative CV; reference and control SEIRAS; gas product analysis; *E*_pzc_ estimation; single-use electrode synthesis and characterization; low-energy ion scattering (LEIS) spectra; inductively coupled plasma results; and supplementary discussion and notes (PDF)

## Abbreviations

CO_2_RR: carbon dioxide reduction reaction
CTAB: cetyltrimethylammonium bromide
CTA^+^: cetyltrimethylammonium
CV: cyclic voltammetry
FE: Faradaic efficiency
HER: hydrogen evolution reaction
RHE: reversible hydrogen electrode
SEIRAS: surface enhanced infrared absorption spectroscopy.

## References

[ref1] Marcandalli G.; Monteiro M. C. O.; Goyal A.; Koper M. T. M. Electrolyte Effects on CO_2_ Electrochemical Reduction to CO. Acc. Chem. Res. 2022, 55 (14), 1900–1911. 10.1021/acs.accounts.2c00080. 35772054 PMC9301915

[ref2] Ringe S. The importance of a charge transfer descriptor for screening potential CO_2_ reduction electrocatalysts. Nat. Commun. 2023, 14 (1), 2598 10.1038/s41467-023-37929-4. 37147278 PMC10162986

[ref3] Cave E. R.; Montoya J. H.; Kuhl K. P.; Abram D. N.; Hatsukade T.; Shi C.; Hahn C.; No̷rskov J. K.; Jaramillo T. F. Electrochemical CO_2_ reduction on Au surfaces: mechanistic aspects regarding the formation of major and minor products. Phys. Chem. Chem. Phys. 2017, 19 (24), 15856–15863. 10.1039/C7CP02855E. 28585950

[ref4] Monteiro M. C. O.; Dattila F.; Hagedoorn B.; García-Muelas R.; López N.; Koper M. T. M. Absence of CO_2_ electroreduction on copper, gold and silver electrodes without metal cations in solution. Nat. Catal. 2021, 4 (8), 654–662. 10.1038/s41929-021-00655-5.

[ref5] Sassenburg M.; Kelly M.; Subramanian S.; Smith W. A.; Burdyny T. Zero-Gap Electrochemical CO_2_ Reduction Cells: Challenges and Operational Strategies for Prevention of Salt Precipitation. ACS Energy Lett. 2023, 8 (1), 321–331. 10.1021/acsenergylett.2c01885. 36660368 PMC9841607

[ref6] Garg S.; Xu Q.; Moss A. B.; Mirolo M.; Deng W.; Chorkendorff I.; Drnec J.; Seger B. How alkali cations affect salt precipitation and CO_2_ electrolysis performance in membrane electrode assembly electrolyzers. Energy Environ. Sci. 2023, 16 (4), 1631–1643. 10.1039/D2EE03725D.

[ref7] Qin H.-G.; Du Y.-F.; Bai Y.-Y.; Li F.-Z.; Yue X.; Wang H.; Peng J.-Z.; Gu J. Surface-immobilized cross-linked cationic polyelectrolyte enables CO_2_ reduction with metal cation-free acidic electrolyte. Nat. Commun. 2023, 14 (1), 5640 10.1038/s41467-023-41396-2. 37704616 PMC10499993

[ref8] Weng S.; Toh W. L.; Surendranath Y. Weakly Coordinating Organic Cations Are Intrinsically Capable of Supporting CO_2_ Reduction Catalysis. J. Am. Chem. Soc. 2023, 145 (30), 16787–16795. 10.1021/jacs.3c04769. 37486158

[ref9] Li W.; Yin Z.; Gao Z.; Wang G.; Li Z.; Wei F.; Wei X.; Peng H.; Hu X.; Xiao L.; et al. Bifunctional ionomers for efficient co-electrolysis of CO_2_ and pure water towards ethylene production at industrial-scale current densities. Nat. Energy 2022, 7 (9), 835–843. 10.1038/s41560-022-01092-9.

[ref10] Fan J.; Pan B.; Wu J.; Shao C.; Wen Z.; Yan Y.; Wang Y.; Li Y. Immobilized Tetraalkylammonium Cations Enable Metal-free CO_2_ Electroreduction in Acid and Pure Water. Angew. Chem. Int. Ed. 2024, 63 (9), e202317828 10.1002/anie.202317828. 38165224

[ref11] Fan M.; Huang J. E.; Miao R. K.; Mao Y.; Ou P.; Li F.; Li X.-Y.; Cao Y.; Zhang Z.; Zhang J.; et al. Cationic-group-functionalized electrocatalysts enable stable acidic CO_2_ electrolysis. Nat. Catal. 2023, 6 (9), 763–772. 10.1038/s41929-023-01003-5.

[ref12] Seh Z. W.; Kibsgaard J.; Dickens C. F.; Chorkendorff I.; No̷rskov J. K.; Jaramillo T. F. Combining theory and experiment in electrocatalysis: Insights into materials design. Science 2017, 355 (6321), eaad4998 10.1126/science.aad4998. 28082532

[ref13] Ortiz-Castillo J. E.; Gallo-Villanueva R. C.; Madou M. J.; Perez-Gonzalez V. H. Anisotropic gold nanoparticles: A survey of recent synthetic methodologies. Coord. Chem. Rev. 2020, 425, 213489 10.1016/j.ccr.2020.213489.

[ref14] Jana N. R.; Gearheart L.; Murphy C. J. Seed-Mediated Growth Approach for Shape-Controlled Synthesis of Spheroidal and Rod-like Gold Nanoparticles Using a Surfactant Template. Adv. Mater. 2001, 13 (18), 1389–1393. 10.1002/1521-4095(200109)13:18<1389::AID-ADMA1389>3.0.CO;2-F.

[ref15] Wu Y.; Yuan X.; Tao Z.; Wang H. Bifunctional electrocatalysis for CO_2_ reduction via surface capping-dependent metal–oxide interactions. Chem. Commun. 2019, 55 (60), 8864–8867. 10.1039/C9CC02934F. 31231725

[ref16] Tao Z.; Wu Z.; Wu Y.; Wang H. Activating Copper for Electrocatalytic CO_2_ Reduction to Formate via Molecular Interactions. ACS Catal. 2020, 10 (16), 9271–9275. 10.1021/acscatal.0c02237.

[ref17] Banerjee S.; Han X.; Thoi V. S. Modulating the Electrode–Electrolyte Interface with Cationic Surfactants in Carbon Dioxide Reduction. ACS Catal. 2019, 9 (6), 5631–5637. 10.1021/acscatal.9b00449.

[ref18] Zhang Z.-Q.; Banerjee S.; Thoi V. S.; Shoji Hall A. Reorganization of Interfacial Water by an Amphiphilic Cationic Surfactant Promotes CO_2_ Reduction. J. Phys. Chem. Lett. 2020, 11 (14), 5457–5463. 10.1021/acs.jpclett.0c01334. 32524821

[ref19] Banerjee S.; Zhang Z.-Q.; Hall A. S.; Thoi V. S. Surfactant Perturbation of Cation Interactions at the Electrode–Electrolyte Interface in Carbon Dioxide Reduction. ACS Catal. 2020, 10 (17), 9907–9914. 10.1021/acscatal.0c02387.

[ref20] Zhu Q.; Murphy C. J.; Baker L. R. Opportunities for Electrocatalytic CO_2_ Reduction Enabled by Surface Ligands. J. Am. Chem. Soc. 2022, 144 (7), 2829–2840. 10.1021/jacs.1c11500. 35137579

[ref21] Pimlott D. J. D.; Jewlal A.; Mowbray B. A. W.; Berlinguette C. P. Impurity-Resistant CO_2_ Reduction Using Reactive Carbon Solutions. ACS Energy Lett. 2023, 8 (4), 1779–1784. 10.1021/acsenergylett.3c00133.

[ref22] Banerjee S.; Gerke C. S.; Thoi V. S. Guiding CO2RR Selectivity by Compositional Tuning in the Electrochemical Double Layer. Acc. Chem. Res. 2022, 55 (4), 504–515. 10.1021/acs.accounts.1c00680. 35119260

[ref23] Chen L.; Li F.; Zhang Y.; Bentley C. L.; Horne M.; Bond A. M.; Zhang J. Electrochemical Reduction of Carbon Dioxide in a Monoethanolamine Capture Medium. ChemSusChem 2017, 10 (20), 4109–4118. 10.1002/cssc.201701075. 28799204

[ref24] Teh W. J.; Kolb M. J.; Calle-Vallejo F.; Yeo B. S. Enhanced Charge Transfer Kinetics for the Electroreduction of Carbon Dioxide on Silver Electrodes Functionalized with Cationic Surfactants. Adv. Funct. Mater. 2023, 33 (7), 2210617 10.1002/adfm.202210617.

[ref25] Sarkar S.; Maitra A.; Banerjee S.; Thoi V. S.; Dawlaty J. M. Electric Fields at Metal–Surfactant Interfaces: A Combined Vibrational Spectroscopy and Capacitance Study. J. Phys. Chem. B 2020, 124 (7), 1311–1321. 10.1021/acs.jpcb.0c00560. 31985221

[ref26] Gisbert-González J. M.; Oliver-Pardo M. V.; Briega-Martos V.; Feliu J. M.; Herrero E. Charge effects on the behavior of CTAB adsorbed on Au(111) electrodes in aqueous solutions. Electrochim. Acta 2021, 370, 137737 10.1016/j.electacta.2021.137737.

[ref27] Banerji L. C.; Jang H.; Gardner A. M.; Cowan A. J. Studying the cation dependence of CO_2_ reduction intermediates at Cu by in situ VSFG spectroscopy. Chem. Sci. 2024, 15 (8), 2889–2897. 10.1039/D3SC05295H. 38404396 PMC10882457

[ref28] Gisbert-González J. M.; Briega-Martos V.; Vidal-Iglesias F. J.; Cuesta Á.; Feliu J. M.; Herrero E. Spectroelectrochemical Studies of CTAB Adsorbed on Gold Surfaces in Perchloric Acid. Langmuir 2023, 39 (7), 2761–2770. 10.1021/acs.langmuir.2c03226. 36753691 PMC9948534

[ref29] Marcandalli G.; Goyal A.; Koper M. T. M. Electrolyte Effects on the Faradaic Efficiency of CO_2_ Reduction to CO on a Gold Electrode. ACS Catal. 2021, 11 (9), 4936–4945. 10.1021/acscatal.1c00272. 34055454 PMC8154322

[ref30] Zosel J.; Oelßner W.; Decker M.; Gerlach G.; Guth U. The measurement of dissolved and gaseous carbon dioxide concentration. Meas. Sci. Technol. 2011, 22 (7), 072001 10.1088/0957-0233/22/7/072001.

[ref31] Gu J.; Liu S.; Ni W.; Ren W.; Haussener S.; Hu X. Modulating electric field distribution by alkali cations for CO_2_ electroreduction in strongly acidic medium. Nat. Catal. 2022, 5 (4), 268–276. 10.1038/s41929-022-00761-y.

