# Supplementary material for: The Role of Surfactant in Electrocatalytic Carbon Dioxide Reduction in the Absence of Metal Cations
Source: ACS Electrochem. 2024 Oct 3;1(1):20–4. doi: 10.1021/acselectrochem.4c00040 (PMC11728718; doi:10.1021/acselectrochem.4c00040)
Supplement: Supplementary file 1 — ec4c00040_si_001.pdf [file ec4c00040_si_001.pdf]

Supporting Information

The Role of Surfactant in Electrocatalytic Carbon Dioxide Reduction in the Absence of Metal Cations

Hansaem Jang,† Adrian M. Gardner,†,‡ Lucy J. Walters,† Alex R. Neale,† Laurence J. Hardwick,† and Alexander J. Cowan*,†

†Stephenson Institute for Renewable Energy (SIRE) and the Department of Chemistry, University of Liverpool, Liverpool L69 7ZF, United Kingdom

‡Low Energy Ion Scattering Facility, George Holt Building, University of Liverpool, Brownlow Street, Liverpool L69 3GB, United Kingdom

* acowan@liverpool.ac.uk

Table of Contents

Experimental

Supplementary Figures and Captions

Supplementary Table and Caption

Supplementary Notes

References for Supporting Information

Experimental

Chemicals and materials

Ar (99.998%; BOC)
Au wire (99.95%; Advent Research Materials; as a deposition target)
Calibration gas (2.8% H_2, 3500 ppm CH_4, 2.8% CO, *etc.* in CO_2 balance; CK Isotopes)
CH_4/CO_2 (1% CH_4 in CO_2 balance; BOC)
CO (99.97%; BOC)
CO_2 (99.995%; BOC)
CTAB (≥99%, BioXtra; Sigma-Aldrich)
Dodecyltrimethylammonium bromide (99%, BioXtra; Sigma-Aldrich)
Ethanol (Absolute grade)
He (99.9999%; BOC)
H_2O (18.2 MΩ cm; Deionized using Direct-Q® 3 UV water purification system; Merck)
H_2SO_4 (≥99.999% as metal-basis; Lot# MKCR4784; Aldrich)
Isopropyl alcohol (≥99.5%, HPLC grade; Fisher chemical)
K_2SO_4 (≥99%; Lot# MKCC0891; Sigma-Aldrich)
Pt wire (>99.99%; GoodFellow; as a counter electrode)

Electrolysis cell

To minimize adventitious impurities arising from the leaching of a cell, disposable Nalgene™ containers (polypropylene copolymer, 125 mL, Thermo Scientific™) were used as an electrolysis cell (Figure S1) after rigorous rinsing with deionized H_2O, as well as acid cleaning, whenever needed. The cell lid was printed with nylon polyamide filament using a 3D-printer (Ultimaker^3). To ensure hermetic sealing, all cell components were assembled with O-rings. As a working electrode, a Au disk electrode (2 mm in diameter; CHI101, CH Instruments, Inc.) was used after being mechanically polished using diamond suspensions (MetaDi™ Supreme, Buehler). The polishing was carried out with the figure-of-8 method on MicroCloth (40-7222, Buehler) in a stepwise manner, starting with 3 μm particle suspensions, followed by 1, 0.25 and 0.05 μm suspensions, respectively. Before and after each polishing step, the electrode was rinsed with H_2O and then sonicated in H_2O. As a counter electrode, Au wire was used after being combined with a glass tube using a septum. As a reference electrode, Pt wire in 0.5 M H_2SO_4 contained in a double-junction chamber (013597 & 013375, ALS Co., Ltd) was used after saturating the filling solution with H_2 by conducting electrolysis at −3 V against Pt auxiliary electrode for the time required for 2 C of charge flowing. The reference electrode was freshly made before experiments. As a neat electrolyte, 1 mM H_2SO_4 solution (pH 2.73) was used without adding any additives to it. For comparative experiments, electrolytes containing CTAB (1 mM) or K_2SO_4 (100 nM, 0.5 mM or 1 mM), in 1 mM H_2SO_4 solution, were prepared. The level of adventitious impurities in the prepared electrolytes was quantified using inductively coupled plasma mass spectrometry (NexION® 2000, PerkinElmer Inc.). Prior to experiments, electrolytes were purged by and saturated with either Ar, CO, CH_4/CO_2 or CO_2 at 20 $cm^3\ min^{-1}$ for at least 30 minutes. In CH_4/CO_2 gas, 1% concentration of CH_4 in CO_2 balance served as an internal standard. Regardless of the saturation gas used (Ar or CO_2) or the presence of 1 mM CTAB or 1 mM K_2SO_4, solutions of 1 mM H_2SO_4 fell under a pH range between 2.7 and 2.8 (HI 83141, Hanna Instruments). Electroanalytical results were converted to an RHE scale with the consideration of the pH of the electrolyte used.

Electroanalysis

A potentiostat (SP200, BioLogic) was used to monitor electroanalytical signals. Unless otherwise noted, the signals were *iR*-corrected with a 100% compensation of the uncompensated resistance (R_u), and R_u was obtained using potentiostatic electrochemical impedance spectroscopy at the open circuit potential (OCP). The electrolyte was kept quiescent throughout measurements. Before or after each measurement, if needed, the electrolyte was agitated to remove bubbles from the electrode surface using a magnetic stirrer bar (20 mm long and 7 mm diameter) and a stirrer plate (Guardian™ 5000, OHAUS). CVs were obtained in various electrolytes (with or without additives) on Au electrode under different gas saturation conditions (Ar, CO or CO_2) at 50 mV s^{-1}. In the initial forward scan, the electrode potential was swept negatively from the OCP to the potential minimum (−1.2 V vs. RHE), located at a potential negative of the typical CO_2RR onset potential of the Au catalyst. In the subsequent reverse scan, the potential was swept positively to the potential maximum (1.3 V vs. RHE), located at a potential positive of the typical CO oxidation potential (i.e. CO-stripping potential). Subsequently, the CV was completed by sweeping the potential back to the OCP. Another electroanalytical technique used in this study was staircase potentiostatic electrochemical impedance spectroscopy (SPEIS). It was conducted in Ar-saturated 1 mM H_2SO_4 electrolyte as a function of applied potential in stepwise decrement (for forward negative scan) or increment (for reverse positive scan) of 10 mV with a hold of 30 s at each potential step. The areal capacitance in series was measured at the set frequency of 1000 Hz at the end of each step. SPEIS was performed also at different frequencies (100, 20 and 10 Hz), and the E_{pzc} was estimated at *ca.* 0.2 V *vs*. RHE.

Gas chromatography (GC)

Bulk electrolysis was carried out in 100 mL of CH_4/CO_2-saturated electrolytes (with or without CTAB and/or K^+ additives) on bare Au electrode using chronopotentiometry at −10 mA cm^{-2} for 3600 s with magnetic stirring at 600 rpm. For comparative experiments to disprove the electrolytic decomposition of CTAB, electrolysis was conducted in Ar-saturated 1 mM CTAB electrolyte in the absence of any internal standards such as CH_4. A leakage correction factor was estimated by averaging the values obtained from all CH_4/CO_2 experiments, and it was applied to the Ar experiment. Using He as the carrier gas, GC (6890N G1530A, Agilent) monitored the gas products, as well as the concentration of the CH_4 internal standard. Calibration curves were obtained to quantify CH_4, CO and H_2. All electrolysis experiments were triplicated.

Low-energy ion scattering (LEIS)

Single-use electrodes were prepared (Figure S11) by growing Au film on polypropylene copolymer sheet (201-2785, RS Components Ltd.) using a thermal evaporator (UNIVEX 300, Leybold). Prior to the deposition, the plastic substrate was sonicated in isopropyl alcohol and dried completely. Au thickness on the substrate was monitored using a quartz crystal microbalance during the deposition. Sheet resistance (R_s) of single-use electrodes was estimated using four-point probe measurements (CMT-SERIES, Advanced Instrument Technology). For R_u estimation, a single-use electrode was assembled in a front-contact sample holder (H-A-S_HOLD-15x15-8dia, redox.me) and served as a working electrode. Then, R_u was obtained at the OCP in 70 mL of Ar-saturated 0.5 M H_2SO_4 under quiescent conditions. Both R_s and R_u values (Figure S12, S13) started to plateau when the Au thickness was 50 nm. On the basis of this observation, electrolysis was conducted using a

single-use 50-nm-Au electrode in CO_2-saturated 1 mM CTAB electrolyte (in 1 mM H_2SO_4) at −1.2 V vs. RHE (without *iR* compensation) for 3600 s with magnetic stirring at 600 rpm. Low Energy Ion Scattering measurements were employed for surface-sensitive characterization a single-use 50-nm-Au electrode before and after electrolysis. A commercial LEIS spectrometer, Qtac[100] (IonTOF) was used for all measurements and analysis was performed in SurfaceLab 7 (IonTOF). In order to observe all surface species present (with an atomic mass ≥ C) a 3 keV He^+ LEIS (ion dose ~3.6 x 10^{14} ions cm^{-2}) analysis beam over a 2 x 2 mm area was employed. The sample was then exposed to a 1 keV Ar^+ sputter ion dose over a 3 x 3 mm area, and a 3 keV He^+ LEIS spectrum again obtained. These cycles were repeated resulting in depth dependent LEIS spectra being obtained. Charge compensation was employed to prevent build-up of excess charge throughout the measurement.

Surface-enhanced infrared absorption spectroscopy

For SEIRAS experiments, we utilized a cell configuration (Figure S3) different from the cell described in the section *Electrolysis cell* (Figure S1) As a working electrode, a Au-coated Si prism was prepared using the following protocol. A Si prism was polished with 1, 0.25 and 0.05 µm diamond suspensions. Before and after each polishing step, the prism was sonicated in 50% ethanol and pure H_2O, respectively. Thereafter, the prism was dried in an oven at 60°C and then cleaned using a plasma cleaner (ZEPTO, Diener electronic GmbH) under Ar for 1 h. Two layers of Au were deposited onto the dried prism. For the first layer, a mask (13 mm in diameter) was located concentrically to the prism surface (20 mm in diameter), and 20 nm of Au were deposited onto the rim of the surface at 0.01 nm s^{-1}. Before the second deposition, the prism was left overnight in a vacuum until reaching *ca.* 10^{-6} mbar. After removing the mask, 18 nm of Au were deposited onto all over the surface at 0.04–0.05 nm s^{-1}. In contact with Au current collectors, the Au-coated prism was assembled to the cell body made of polyether ether ketone. For both counter and pseudo-reference electrodes, Pt coils were used. The pseudo-reference electrode potential was identified by comparing with a Ag/AgCl master electrode (E-Ag/AgCl_70, redox.me). As an electrolyte, 1 mM H_2SO_4 with or without 1 mM CTAB was used. Prior to electrolysis, electrolytes were saturated with CO_2 outside of the cell chamber and then an aliquot of 1.5 mL was transferred to the chamber. After assembling all cell components together, the headspace was purged out with CO_2. The cell assembly was placed in the sample compartment within a Fourier transform infrared spectrometer (VERTEX 70v, Bruker), fitted with a MCT (mercury cadmium telluride) detector. The angle of incidence was set at 65° and unpolarised IR radiation was used. Spectra were collected over 30 s and consist of 70 coadded interferograms at 4 cm^{-1} resolution. After connecting the electrodes to cables (Figure S3), the spectrometer was sealed and evacuated to a vacuum. A potentiostat (SP50, BioLogic) was employed to apply constant potentials in a stepwise manner in decrement or increment of 0.1 V with a hold of 60 s at each potential step. Each infrared spectrum was obtained 10 s after changing potentials. The spectrum collected at the OCP (0.77 V without CTAB and 0.31 V with CTAB) was used as a background spectrum. Despite the vacuum sample chamber, ro-vibrational lines assigned to the bending vibration of atmospheric water were observed in the SEIRAS spectra; using a reference air spectrum (Figure S4), the SEIRAS spectra were fitted to remove baseline with a scaling and shifting factor. The spectra were plotted with the relative change in infrared signals (ΔS) with respect to the signals obtained at the OCP (S_{OCP}), i.e. $\Delta S/S_{OCP}$ (or $\Delta S/S$) where $\Delta S = -(S_{variable} - S_{OCP})$, and $S_{variable}$ is the infrared signals obtained under biased conditions (Figure 2, Figure S5) or varied atmospheric conditions (Figure S4).

Supplementary Figures and Captions

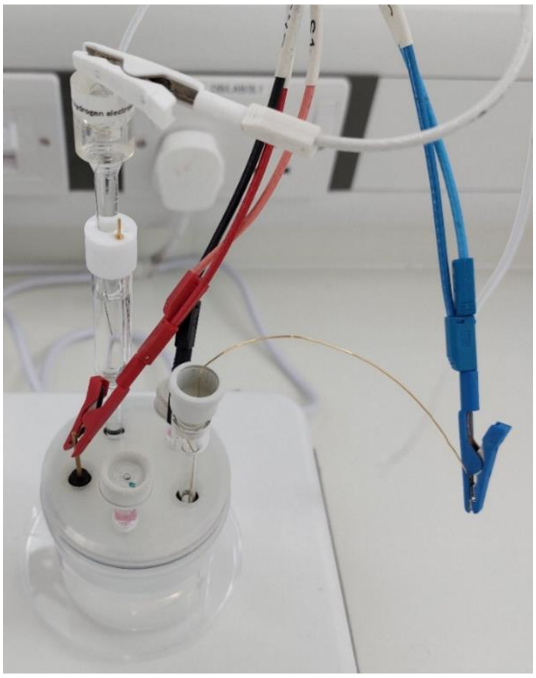

Figure S1. Photograph of a cell set-up employed for electroanalysis and bulk electrolysis. Red, white and blue alligator clips are connected to working, reference and counter electrodes, respectively.

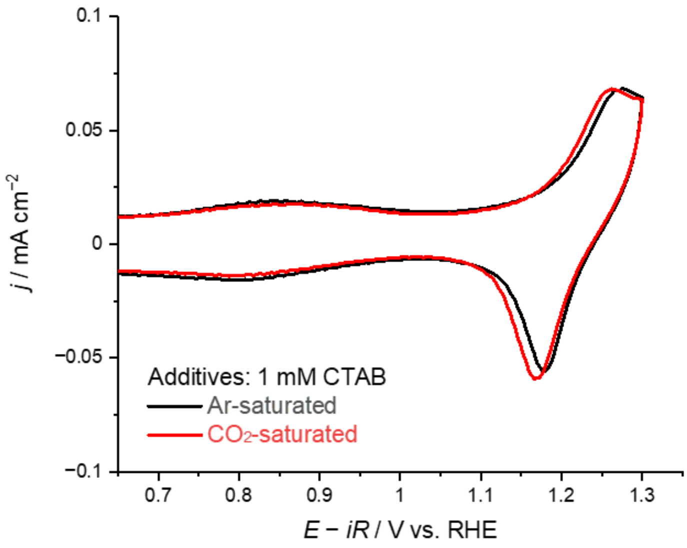

Figure S2. Magnified cyclic voltammograms obtained in the 1 mM H_2SO_4 and 1 mM CTAB electrolyte within a potential window relating to CO-stripping. The electrolyte was purged and saturated with (Black) Ar or (Red) CO_2. The electrode potential was initially scanned from the OCP to −1.2 V (potentials negative of CO evolution), then to 1.3 V, and back to the OCP at 50 mV s^{-1}.

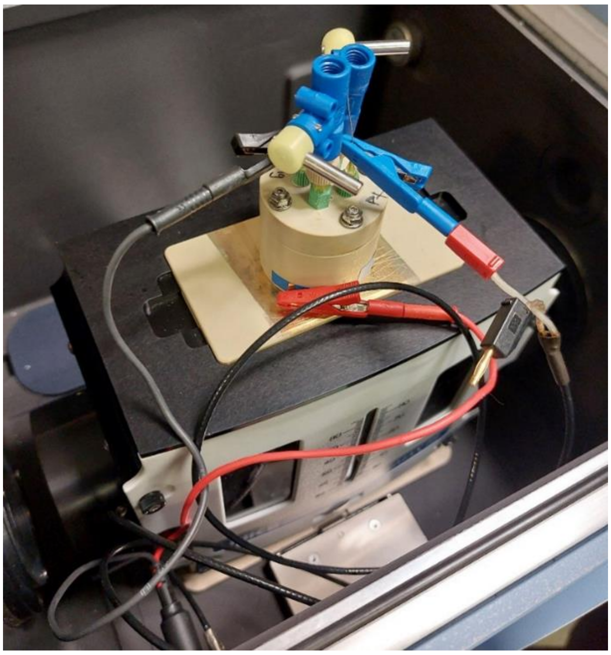

Figure S3. Photograph of a surface-enhanced infrared absorption spectroscopy cell placed inside the spectrometer compartment. Red, black and blue alligator clips are connected to working, pseudo-reference and counter electrodes, respectively. The cell was sealed before performing experiments. Cross-sectional schematic diagram of a spectroelectrochemical cell configuration can be found elsewhere.[1,2]

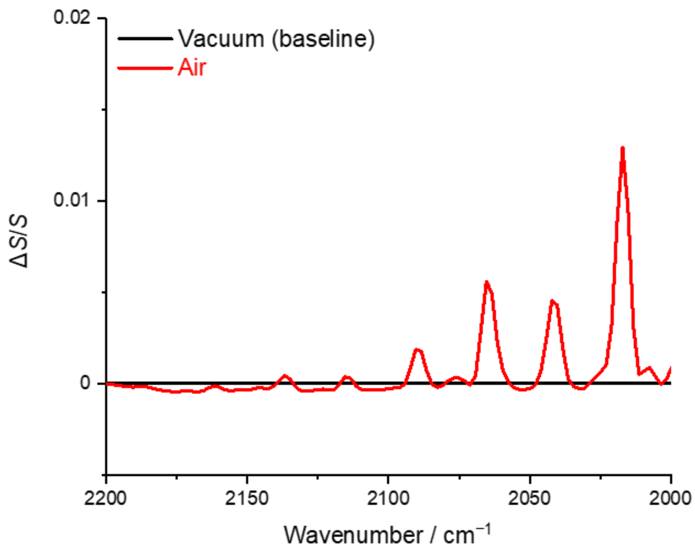

Figure S4. Ro-vibrational lines assigned to the bending vibration of atmospheric water, air infrared spectrum.

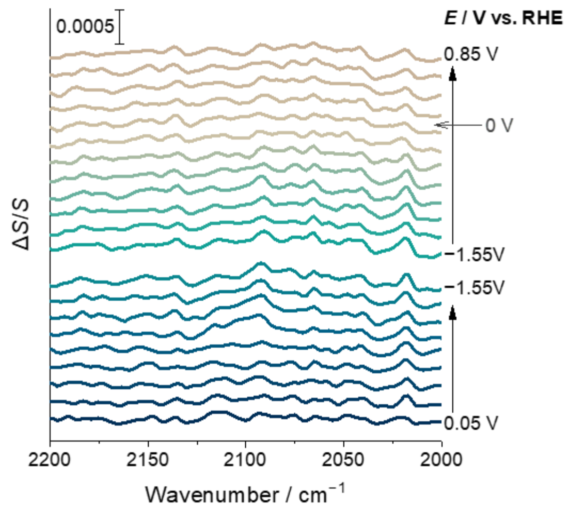

Figures S5. *Operando* attenuated total reflectance surface-enhanced infrared absorption spectroscopy spectra obtained in 1 mM H_2SO_4 electrolyte saturated with CO_2 as a function of varied applied potential in stepwise decrement or increment of 0.2 V.

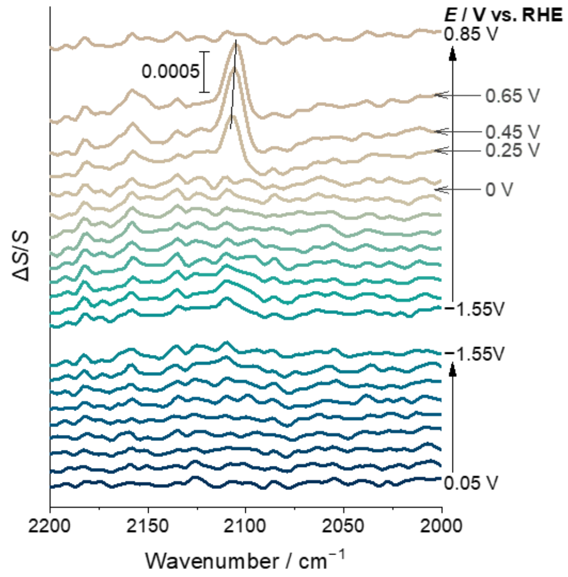

Figure S6. Figure 2 with a line along the CO_{ad} peak maxima to guide the eye. *Operando* SEIRAS spectra obtained in the 1 mM H_2SO_4 and 1 mM CTAB electrolyte saturated with CO_2, as a function of varied applied potential in stepwise decrement or increment of 0.2 V.

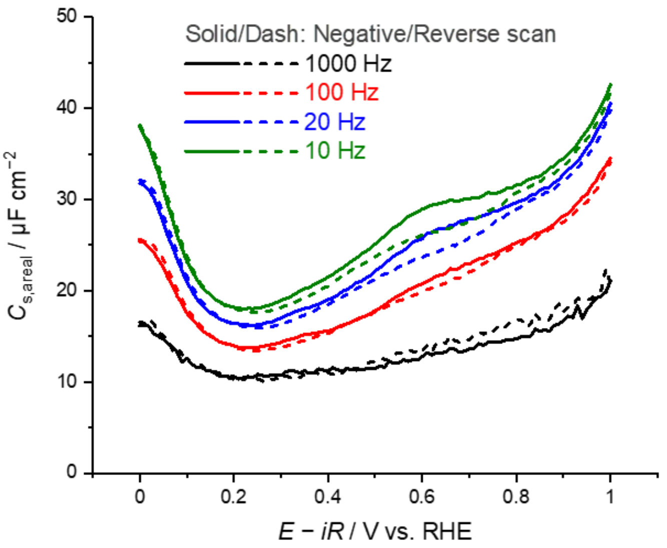

Figure S7. Areal capacitance calculated on the basis of data obtained from staircase potentiostatic electrochemical impedance spectroscopy on Au in 1 mM H_2SO_4 as a function of applied potential.

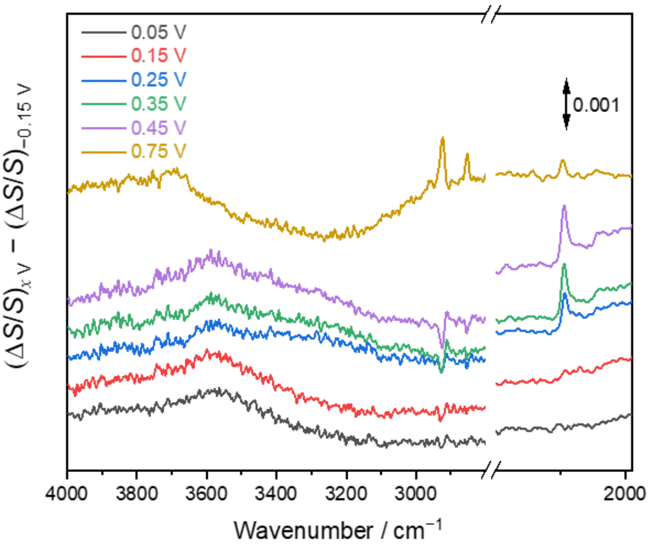

Figure S8. *Operando* SEIRAS spectra obtained in 1 mM H_2SO_4 and 1 mM CTAB electrolyte saturated with CO_2. During the experiment the gold electrode potential is changed from open circuit to –1.55 V (a potential where CO_2 reduction occurs) and back to +0.85 V. Figure S8 covers the potential window of 0.05 V to 0.75 V as the potential is increased with the SEIRAS spectra referenced to the spectra recorded at –0.15 V (a potential negative of the potential of zero charge). Figure S8 highlights the change in electrode surface as the electrode is made positive of the potential of zero charge.

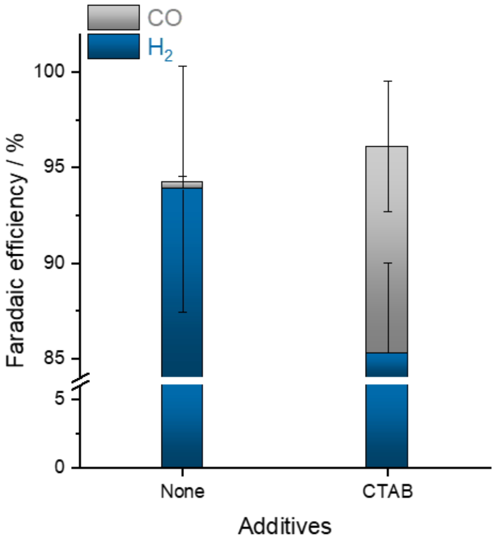

Figure S9. Composition analysis of gas products after bulk electrolysis using chronopotentiometry at −10 mA cm^{-2} for 3600 s with magnetic stirring at 600 rpm in CO_2-saturated electrolytes with or without 1 mM CTAB in 1 mM H_2SO_4 solution. The averaged iR-corrected applied potential to achieve the current density was −1.05±0.23 V in the absence of CTAB and −1.30±0.16 V in the presence of CTAB. Methane as an internal standard was present in the cell with a concentration of 1% in CO_2 balance.

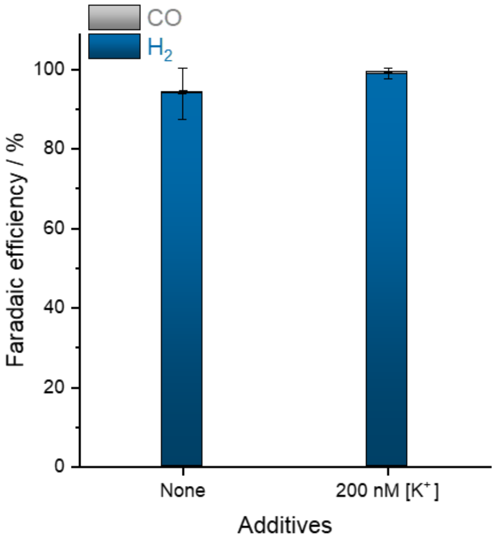

Figure S10. Composition analysis of gas products after bulk electrolysis in CO_2-saturated electrolytes with or without 200 nM [K^+] (i.e. 100 nM K_2SO_4) in 1 mM H_2SO_4 solution. Methane as an internal standard was present in the cell with a concentration of 1% in CO_2 balance.

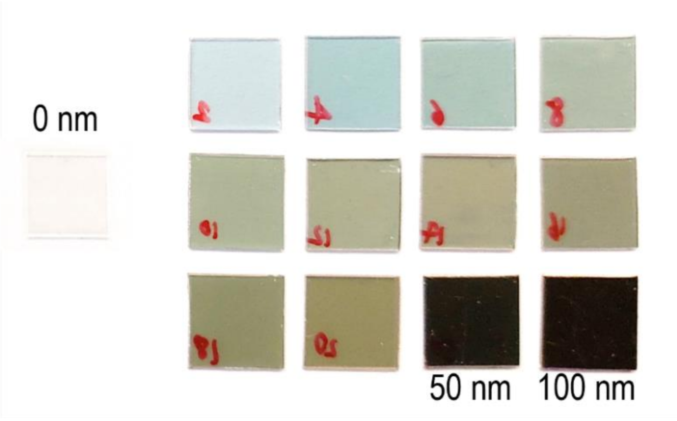

Figure S11. Photographs of plastic substrates with varied Au deposition thickness (0, 2, 4, 6, 8, 10, 12, 14, 16, 18, 20, 50, 100 nm).

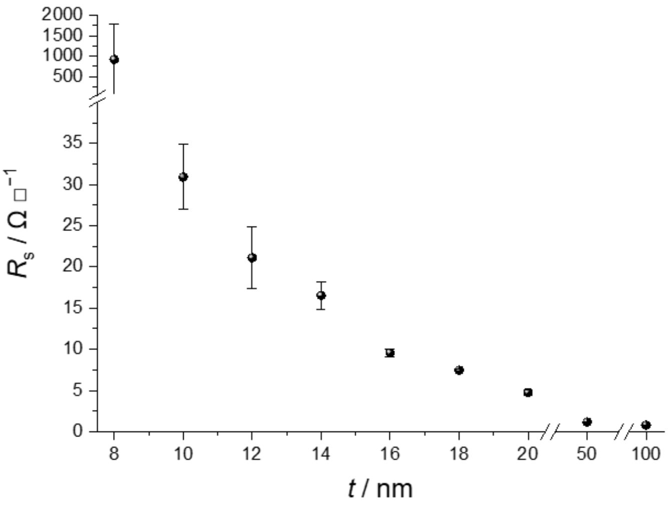

Figure S12. Sheet resistance of single-use Au electrodes as a function of Au thickness.

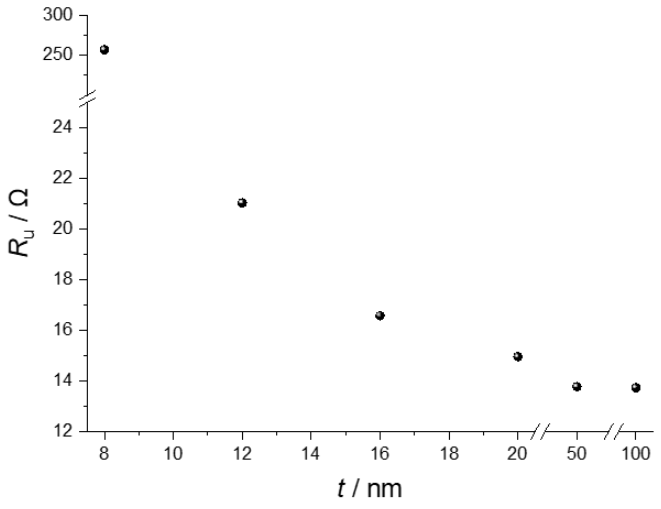

Figure S13. Uncompensated resistance of single-use Au electrodes obtained in 0.5 M H_2SO_4 electrolyte as a function of Au thickness.

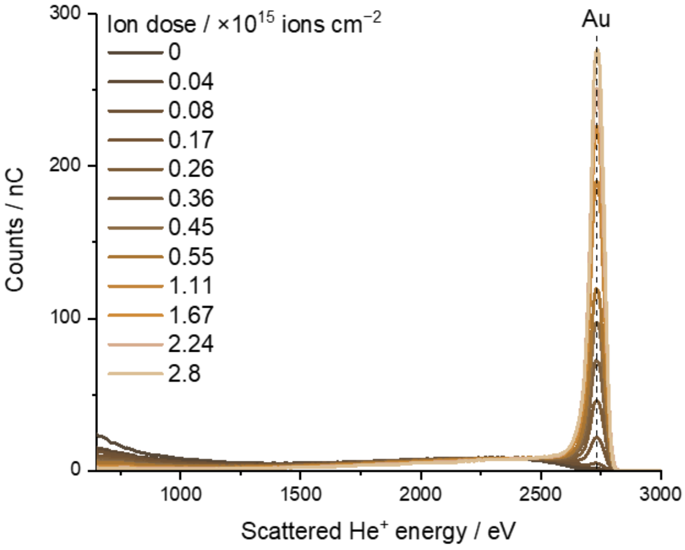

Figure S14. 3 keV He^+ low-energy ion scattering spectra of a single-use 50-nm-Au electrode before electrolysis, obtained after exposure to a 1 keV Ar^+ sputter ion dose as indicated in the legend.

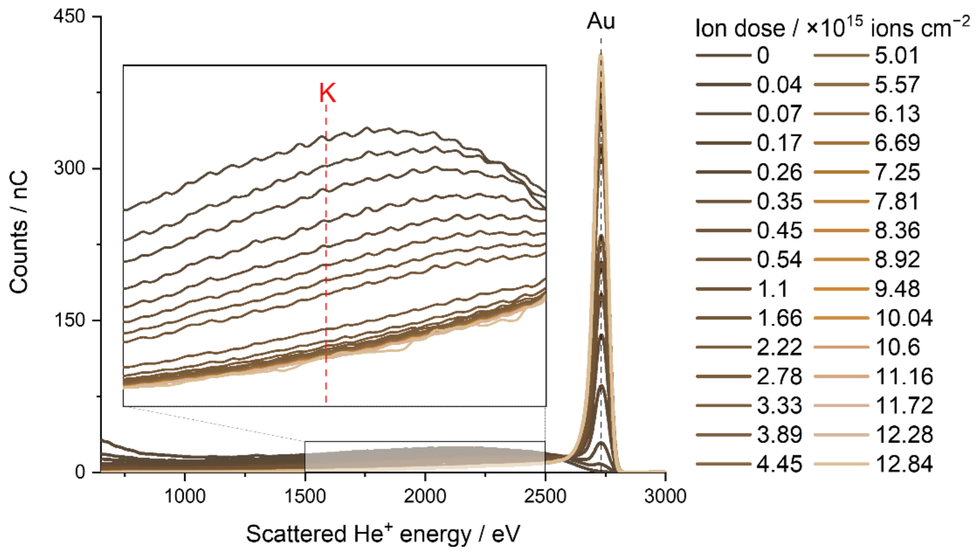

Figure S15. 3 keV He^+ low-energy ion scattering spectra of a single-use 50-nm-Au electrode after electrolysis in 1 mM CTAB electrolyte saturated with CO_2, obtained after exposure to a 1 keV Ar^+ sputter ion dose as indicated in the legend, with an inset of magnified spectra marked with an expected K surface peak position by dashed red line. The expected peak position is based on expectations from elastic scattering of He^+ from surface potassium species.[3,4]

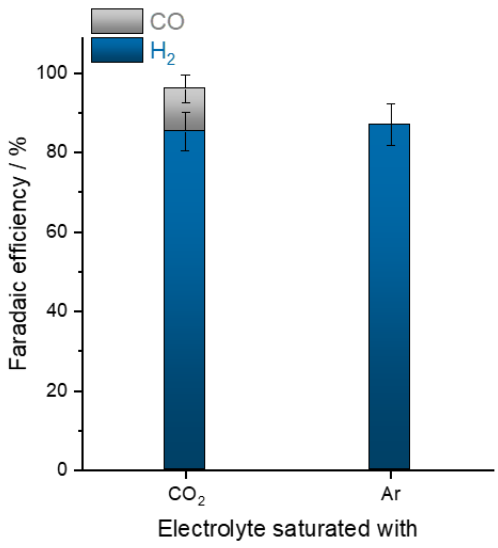

Figure S16. Composition analysis of gas products after bulk electrolysis in the 1 mM H_2SO_4 and 1 mM CTAB electrolyte saturated with CO_2RR-relavant or CO_2RR-irrelvant gas. For the CO_2 experiment, CH_4 as an internal standard was present in the cell with a concentration of 1% in CO_2 balance. For the Ar experiment, any gases other than Ar were not provided to the cell.

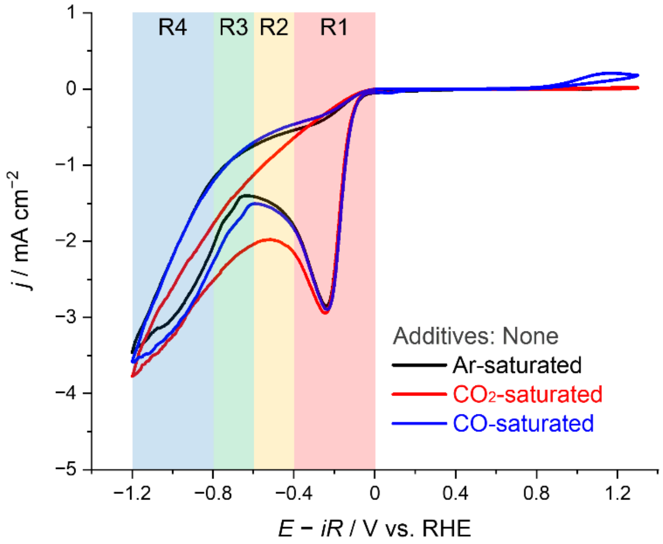

Figure S17. Cyclic voltammograms obtained in 1 mM H_2SO_4 solution, saturated with Ar, CO_2 or CO, as specified in the legend. The electrode potential was initially scanned from the OCP to −1.2 V, then to 1.3 V, and back to the OCP at 50 mV s^{-1}.

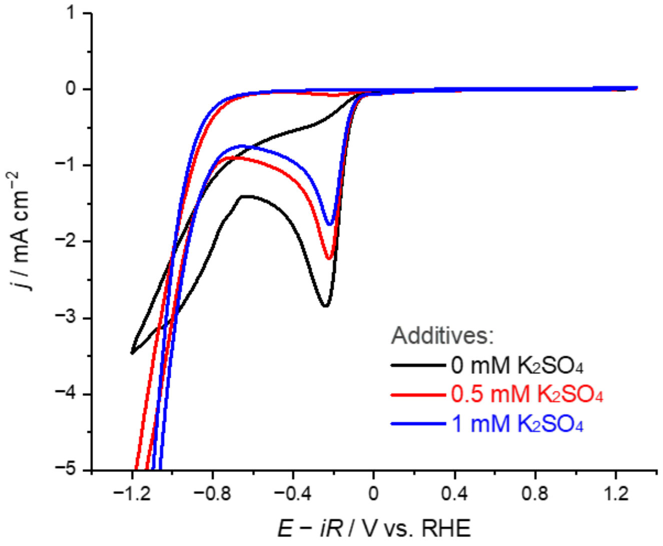

Figure S18. Cyclic voltammograms obtained in Ar-saturated electrolytes with or without K_2SO_4 addition. The electrode potential was initially scanned from the OCP to −1.2 V, then to 1.3 V, and back to the OCP at 50 mV s^{-1}.

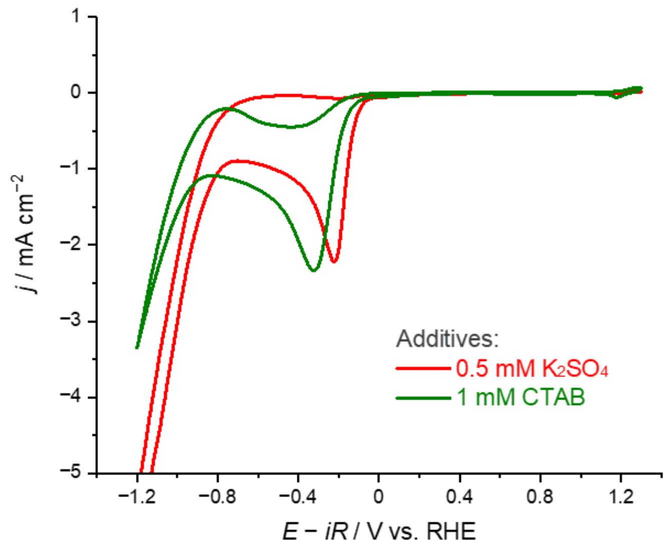

Figure S19. Cyclic voltammograms obtained in Ar-saturated electrolytes, either a 0.5 mM K_2SO_4 and 1 mM H_2SO_4 solution or a 1 mM CTAB and 1 mM H_2SO_4 solution. The electrode potential was initially scanned from the OCP to −1.2 V, then to 1.3 V, and back to the OCP at 50 mV s^{-1}.

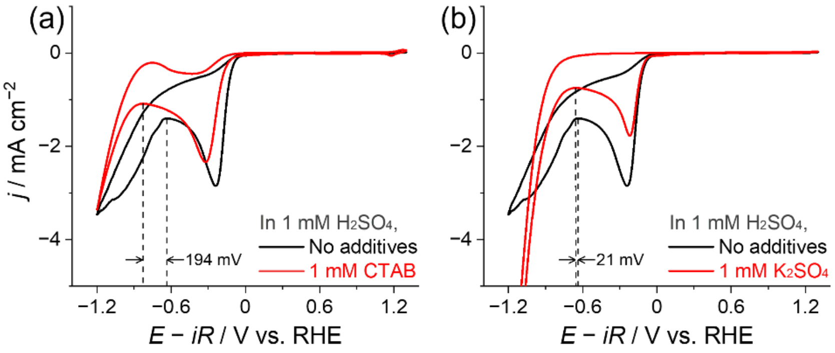

Figure S20. Figure 4 with a different H_2O/H_2 onset potential estimation method. Cyclic voltammograms obtained on Au in Ar-saturated electrolytes, with or without (a) 1 mM CTAB or (b) 1 mM K_2SO_4 in the 1 mM H2SO4 solution. The electrode potential was initially scanned from the OCP to −1.2 V, then to 1.3 V, and back to the OCP at 50 mV s^{-1}.

Supplementary Table and Caption

Table S1. Alkali metal impurities existing in the solutions used in this study.

	Li	Na	K	Cs
Blank[a]	N/D[b]	N/D[b]	N/D[b]	N/D[b]
1 mM H_2SO_4	N/D[b]	N/D[b]	N/D[b]	N/D[b]
1 mM H_2SO_4 + 1 mM CTAB (before electrolysis)	N/D[b]	N/D[b]	191.92 nM	N/D[b]
1 mM H_2SO_4 + 1 mM CTAB (after electrolysis)[c]	N/D[b]	67.72 nM	116.19 nM	N/D[b]

[a] Deionized H_2O. [b] Not detected as it is below the detection limit; the detection limit is 1 ppb for all measured elements, i.e. 114.1, 43.5, 25.6, 7.5 nM for Li, Na, K and Cs, respectively. [c] The total alkali metal cation concentration is below 200 nM, which is lower than a control experiment with intentionally added alkali metal impurities of 200 nM (Figure S10).

Supplementary Notes

Note S1. A potential-dependent shift (PDS) is calculated based on the peak position shift between potentials at 0.25 V (2106.5 cm^{-1}) and 0.65 V (2105.3 cm^{-1}), which gives a PDS of -3 cm^{-1} V^{-1} (Figure S6). Interestingly, the obtained PDS is much smaller than values reported in the past studies conducted in the presence of metal cations, where a PDS is 24 cm^{-1} V^{-1} (calculated from the CO_2RR–SEIRAS spectra on Au in 500 mM $NaHCO_3$)[5] or 24.3±0.6 cm^{-1} V^{-1} (measured using vibrational sum frequency generation spectroscopy on Au in 100 mM $NaHCO_3$ prepared from 50 mM Na_2CO_3 solution)[6]. It suggests that the interfacial electric field strength,[7–9] and/or surface coverage,[10] can be changed and controlled by replacing the metal cation with CTAB in the electrolyte.

Note S2. Figure S8 shows that at potentials close to and positive of the potential of zero charge (+0.05 V to 0.45 V) there is a decrease in the intensity of the ν(CH) modes assignable to CTA^+ on the electrode surface at 2923 and 2852 cm^{-1}.[11] At these potentials we also find an increase in the ν(OH) modes of the interfacial water at 3200, 3400 and 3600 cm^{-1}. This is in line with past studies that also reported water access to the Au surface increases at potentials positive of zero charge. The loss of the CTA^+ and the increased water at the Au surface coincides with the observation of CO_{ad} in the SEIRAS spectra (*ca.* 2090 cm^{-1}; +0.05 V to 0.45 V). At these potentials CO_2 reduction does not occur and the CO measured in the SEIRAS experiment arises from CO accumulated in the electrolyte when the electrode was held at more negative potentials. Taken together these experiments show (i) reordering/removal of the positively charge CTA^+ is required to enable CO_{ad} to be present at significant quantities and (ii) that CTA^+ at the electrode surface also hampers H_2O access to the Au surface in-line with our electroanalytical studies in the main text. Once CO_{ads} is stripped from the surface at the most positive potentials (0.75 V) we see an increase in the intensity of the CTA^+ ν(CH) modes and a displacement of water from the electrode surface.

Note S3. We investigate the possibility that CO evolves as a result of adventitious impurities. We identified the content of alkali metal cations in the solutions of interest, using inductively coupled plasma mass spectrometry (Table S1). 192 nM of K^+ ions existed in 1 mM CTAB solution, so we conducted electrolysis in 200 nM [K^+] solution devoid of CTAB. No CO_2RR was detected by either the electroanalytical method (Figure 1b) or by GC (Figure S10) in the 200 nM [K^+] experiment. Moreover, we were unable to detect K on the single-use Au electrode (characterised Figure S11–S13) after the CTAB experiment, using low-energy ion scattering spectra (Figure S14, S15), a highly sensitive surface analysis technique. This demonstrates that the metal cation impurities are unlikely to be responsible for the CO production observed in the CTAB experiment.

Note S4. We investigate the possibility that CO evolves as a result of the electrolytic decomposition of CTA^+. However, electrolysis performed in 1 mM CTAB electrolyte saturated with Ar did not produce any CO (Figure S16). Therefore, we conclude that CO production in the absence of alkali cations can arise from the CO_2RR, enabled by the presence of CTA^+ at the electrode surface.

Note S5. The only experimental difference between Figure 1c Black and Red is the type of saturation gas used to purge and saturate the electrolyte. The feature in R2 appears when CO_2 is used as a saturation gas; therefore, it is reasonable to assign this feature to possible derivatives of CO_2 in the electrolyte system employed (i.e. 1 mM H_2SO_4 solution devoid of further additives in Figure 1c). For instance, as discussed in the main text, HCO_3^- due to a shift in local pH can be produced from CO_2 in H_2O. Albeit unlikely to occur, if CO_2 is electrochemically reduced at the R1 potential window (i.e. between 0 and −0.4 V), CO can be generated, which can be considered as another derivative of CO_2. To investigate whether CO is responsible for the R2 signature, we prepared an electrolyte saturated with CO and performed electrolysis (Figure S17). When comparing it with the Ar-saturated experiment, a good overlap in current density is seen throughout the R2 potential window, indicating that CO cannot account for the increase in current observed when CO_2 is selected as a saturation gas. Consequently, we assigned the R2 signature as the electroreduction of HCO_3^-, rather than CO. HCO_3^- formation is possible despite the low bulk pH in R2 as H^+ reduction in R1 will lead to a rise in local pH at the electrode.

Note S6. It is worthwhile to note that the shielding effect depends primarily on the cation concentration (c_{cation}).[12] When the c_{K+} of the electrolyte decreases from 2 mM to 1 mM, the limiting current increases to −2.22 mA cm^{-2} (Figure S18). This value is comparable to that of 1 mM CTAB, i.e. −2.33 mA cm^{-2} (Figure 3).

Note S7. If H^+ transport to the electrode surface is hindered by the shielding effect, the onset potential for H^+/H_2 should also be accordingly shifted to more negative potentials. Figure 3 inset shows that 1 mM of CTAB in solution shifted the H^+/H_2 onset potential by −70 mV, when compared to the 1 mM H_2SO_4 electrolyte alone. Here we define the H^+/H_2 onset potential as the potential where the current density reaches −1 mA cm^{-2} in R1. A shift of −13 mV is also measured in the 1 mM H_2SO_4 and 1 mM K_2SO_4 solution. In both cases, the onset potential was negatively shifted, supporting that the shielding effect can retard the migration of H^+ towards the electrode surface and inhibits the HER. However, 1 mM CTAB, albeit lower in c_{cation}, shifted the onset potential to a greater extent than 1 mM K_2SO_4. This observation demonstrates that the onset potential shift for CTAB cannot be accounted for solely by the shielding effect.

In addition, we estimate c_{cation} values based on bulk concentrations of cations in the electrolyte, assuming the c_{K+} in 1 mM K_2SO_4 as 2 mM [K^+] and the c_{CTA+} in 1 mM CTAB as 1 mM CTA^+. However, the estimated c_{cation} may not represent the actual c_{cation} on the basis of two reasons. First, the critical micelle concentration of CTAB is *ca.* 1 mM in H_2O at room temperature,[13] which can change depending on the solution pH and the identity and concentration of other ions coexisting in the solution. Second, the local c_{cation} at the electrode surface is dependent on the potential applied to the electrode; for example, during electrolysis, positively charged species such as K^+ can be accumulated at the negatively charged electrode.[14,15] Since it is complicated to explicitly estimate the actual c_{cation} of the surface, we additionally compare two experimental results where the strength of the shielding effect is comparable between each other, i.e. 0.5 mM K_2SO_4 versus 1 mM CTAB (Figure S19). Despite the similarity in H^+/H_2 limiting current, the H^+/H_2 onset of 1 mM CTAB is located 64 mV negative of 0.5 mM K_2SO_4 at −1 mA cm^{-2}. It corroborates that the shielding effect alone cannot account for the onset potential shift observed in the CTAB experiment.

Note S8. In the main text, we define the H_2O/H_2 onset potential as the potential when the current density reaches −2 mA cm^{-2} in the forward scan past the H^+/H_2 peak in Ar-saturated electrolyte (i.e. under CO_2-free condition). We chose this value with the following criteria: 1. Current should represent the H_2O/H_2 reaction, 2. The contribution of H^+/H_2 current to the HER should be minimal (i.e. a potential should be located much more negative than H^+/H_2 peak and the consecutive H^+/H_2 plateau currents), 3. The contribution of metal cation effects to H_2O/H_2 HER activity should be minimal (i.e. a potential shouldn't be too negative). We are aware that the aforementioned methodology cannot fully represent the actual H_2O/H_2 onset potential values. Therefore, we also estimated the H_2O/H_2 onset potential using a secondary method (Figure S20). Here, we define the onset potential as the potential at the local current minimum in the forward scan past the H^+/H_2 onset potential in Ar-saturated electrolyte (i.e. under CO_2-free condition). Again, the same conclusion can be drawn in Figure S20 that H_2O/H_2 begins to start at much negative potentials when CTA^+ is introduced to the electrolyte.

Note S9. Thus far, we have only used CTAB as our model cationic surfactant. This is because CTAB is a surfactant that is extensively used for Au nanoparticle synthesis,[16,17] and its removal after synthesis is known to be notoriously difficult.[18] To show that our observation is not unique to CTAB but is imparted by the cationic functionality of surfactant, we further perform electrolysis in the presence of a cationic surfactant having different alkyl chain length instead of CTAB. When the 1 mM dodecyltrimethylammonium bromide was introduced to the 1 mM H_2SO_4 solution, the CO_2RR was also enabled, giving a FE_{H2} of 75.92% and a FE_{CO} of 19.18%.

References for Supporting Information

[1] Vivek, J. P.; Berry, N.; Papageorgiou, G.; Nichols, R. J.; Hardwick, L. J. Mechanistic Insight into the Superoxide Induced Ring Opening in Propylene Carbonate Based Electrolytes using in Situ Surface-Enhanced Infrared Spectroscopy. *J. Am. Chem. Soc.* **2016**, *138* (11), 3745–3751.

[2] Vivek, J. P.; Berry, N. G.; Zou, J.; Nichols, R. J.; Hardwick, L. J. In Situ Surface-Enhanced Infrared Spectroscopy to Identify Oxygen Reduction Products in Nonaqueous Metal–Oxygen Batteries. *J. Phys. Chem. C* **2017**, *121* (36), 19657–19667.

[3] Cushman, C. V.; Brüner, P.; Zakel, J.; Major, G. H.; Lunt, B. M.; Smith, N. J.; Grehl, T.; Linford, M. R. Low energy ion scattering (LEIS). A practical introduction to its theory, instrumentation, and applications. *Anal. Methods* **2016**, *8*, 3419–3439.

[4] Průša, S.; Linford, M. R.; Vaníčková, E.; Bábík, P.; Pinder, J. W.; Šikola, T.; Brongersma, H. H. A practical guide to interpreting low energy ion scattering (LEIS) spectra. *Appl. Surf. Sci.* **2024**, *657*, 158793.

[5] Dunwell, M.; Lu, Q.; Heyes, J. M.; Rosen, J.; Chen, J. G.; Yan, Y.; Jiao, F.; Xu, B. The Central Role of Bicarbonate in the Electrochemical Reduction of Carbon Dioxide on Gold. *J. Am. Chem. Soc*. **2017**, *139* (10), 3774–3783.

[6] Zhu, Q.; Wallentine, S. K.; Deng, G.-H.; Rebstock, J. A.; Baker, L. R. The Solvation-Induced Onsager Reaction Field Rather than the Double-Layer Field Controls CO_2 Reduction on Gold. *JACS Au* **2022**, *2* (2), 472–482.

[7] Gunathunge, C. M.; Ovalle, V. J.; Waegele, M. M. Probing promoting effects of alkali cations on the reduction of CO at the aqueous electrolyte/copper interface. *Phys. Chem. Chem. Phys.* **2017**,*19*, 30166–30172.

[8] Li, H.; Jiang, K.; Zou, S.-Z.; Cai, W.-B. Fundamental aspects in CO_2 electroreduction reaction and solutions from in situ vibrational spectroscopies. *Chin. J. Catal.* **2022**, *43* (11), 2772–2791.

[9] Banerji, L. C.; Jang, H.; Gardner, A. M.; Cowan, A. J. Studying the cation dependence of CO_2 reduction intermediates at Cu by *in situ* VSFG spectroscopy. *Chem. Sci.* **2024**, *15* (8) 2889–2897.

[10] Hollins, P.; Pritchard, J. Infrared studies of chemisorbed layers on single crystals. *Prog. Surf. Sci.* **1985**, *19* (4), 275–349.

[11] Gisbert-González, J. M.; Briega-Martos, V.; Vidal-Iglesias, F. J.; Cuesta, Á; Feliu, J. M.; Herrero, E. Spectroelectrochemical Studies of CTAB Adsorbed on Gold Surfaces in Perchloric Acid. *Langmuir* **2023**, *39* (7) 2761–2770.

[12] Qin, H.-G.; Li, F.-Z.; Du, Y.-F.; Yang, L.-F.; Wang, H.; Bai, Y.-Y.; Lin, M.; Gu, J. Quantitative Understanding of Cation Effects on the Electrochemical Reduction of CO_2 and H^+ in Acidic Solution. *ACS Catal*. **2023**, 13 (2), 916–926.

[13] Zdziennicka, A.; Szymczyk, K.; Krawczyk, J.; Jańczuk, B. Critical micelle concentration of some surfactants and thermodynamic parameters of their micellization. *Fluid Ph. Equilib.* **2012**, *322–323*, 126–134.

[14] Ringe, S.; Morales-Guio, C. G.; Chen, L. D.; Fields, M.; Jaramillo, T. F.; Hahn, C.; Chan, K. Double layer charging driven carbon dioxide adsorption limits the rate of electrochemical carbon dioxide reduction on Gold. *Nat. Commun.* **2020**, *11*, 33.

[15] Zou, X.; Gu, J.; Strategies for efficient CO_2 electroreduction in acidic conditions. *Chin. J. Catal.* **2023**, *52*, 14–31.

[16] Ortiz-Castillo, J. E.; Gallo-Villanueva, R. C.; Madou, M. J.; Perez-Gonzalez, V. H. Anisotropic gold nanoparticles: A survey of recent synthetic methodologies. *Coord. Chem. Rev*. **2020**, *425*, 213489.

[17] Jana, N. R.; Gearheart, L.; Murphy, C. J. Seed-Mediated Growth Approach for Shape-Controlled Synthesis of Spheroidal and Rod-like Gold Nanoparticles Using a Surfactant Template. *Adv. Mater.* **2001**, *13* (18), 1389–1393.

[18] Schulz, F.; Friedrich, W.; Hoppe, K.; Vossmeyer, T.; Weller, H.; Lange, H. Effective PEGylation of gold nanorods. *Nanoscale* **2016**, *8*, 7296–7308.
